# Endogenous dopamine release in the human brain as a pharmacodynamic biomarker: evaluation of the new GPR139 agonist TAK-041 with [^11^C]PHNO PET

**DOI:** 10.1038/s41386-021-01204-1

**Published:** 2021-10-21

**Authors:** Eugenii A. Rabiner, Tolga Uz, Ayla Mansur, Terry Brown, Grace Chen, Jingtao Wu, Joy Atienza, Adam J. Schwarz, Wei Yin, Yvonne Lewis, Graham E. Searle, Jeremy M. T. J. Dennison, Jan Passchier, Roger N. Gunn, Johannes Tauscher

**Affiliations:** 1 Invicro, London, UK; 2 Centre for Neuroimaging Sciences, Institute of Psychiatry, Psychology and Neuroscience, King’s College London, London, UK; 3 Takeda Pharmaceuticals Ltd, Cambridge, MA USA; 4 Hammersmith Medicines Research, London, UK

**Keywords:** Predictive markers, Predictive markers

## Abstract

The use of positron emission tomography (PET) in early-phase development of novel drugs targeting the central nervous system, is well established for the evaluation of brain penetration and target engagement. However, when novel targets are involved a suitable PET ligand is not always available. We demonstrate an alternative approach that evaluates the attenuation of amphetamine-induced synaptic dopamine release by a novel agonist of the orphan G-protein-coupled receptor GPR139 (TAK-041). GPR139 agonism is a novel candidate mechanism for the treatment of schizophrenia and other disorders associated with social and cognitive dysfunction. Ten healthy volunteers underwent [^11^C]PHNO PET at baseline, and twice after receiving an oral dose of d-amphetamine (0.5 mg/kg). One of the post-d-amphetamine scans for each subject was preceded by a single oral dose of TAK-041 (20 mg in five; 40 mg in the other five participants). D-amphetamine induced a significant decrease in [^11^C]PHNO binding potential relative to the non-displaceable component (BP_ND_) in all regions examined (16–28%), consistent with increased synaptic dopamine release. Pre-treatment with TAK-041 significantly attenuated the d-amphetamine-induced reduction in BP_ND_ in the a priori defined regions (putamen and ventral striatum: 26% and 18%, respectively). The reduction in BP_ND_ was generally higher after the 40 mg than the 20 mg TAK-041 dose, with the difference between doses reaching statistical significance in the putamen. Our findings suggest that TAK-041 enters the human brain and interacts with GPR139 to affect endogenous dopamine release. [^11^C]PHNO PET is a practical method to detect the effects of novel drugs on the brain dopaminergic system in healthy volunteers, in the early stages of drug development.

## Introduction

Positron emission tomography (PET) studies have become an essential component of early-phase development for novel small-molecule pharmaceuticals targeting the central nervous system (CNS). No other methods allow direct quantification of the relationship between the concentration of the drug in plasma and target engagement in the human brain. These PET studies typically use a radioligand specific for the molecular target in question and relate changes in target occupancy, as measured with PET at several time points subsequent to a single oral dose of the drug under investigation, to drug concentration in the plasma at the time of the PET scans. Such a study helps to confirm brain penetration and central target engagement and, together with proper modelling approaches, provides a rational basis for drug-dosing schedules in future clinical trials. Data derived from such studies can establish the time–exposure–occupancy relationship for a novel compound [1, 2], enabling the selection of a relevant dose range, thereby significantly increasing efficiency, reducing time and cost, and increasing the probability of success of subsequent clinical trials.

This approach depends on the availability of a suitable PET radioligand for the molecular target in question. Such ligands may not exist for truly novel targets. In this manuscript, we describe an alternative approach that can be useful when a specific radioligand for the molecular target is not available. This approach uses a PET radioligand that is sensitive to the concentration of an endogenous neurotransmitter to detect changes in endogenous neurotransmitter levels induced by the novel pharmaceutical.

G-protein-coupled receptor 139 (GPR139) is a novel, highly conserved, class A orphan receptor [3, 4] belonging to the gamma-rhodopsin family. GPR139 is expressed in the medial habenula, septum, hypothalamus, basal ganglia, thalamus and midbrain dopaminergic nuclei [5–7]. The CNS localisation of this receptor suggests its potential contribution to movement and metabolism, with a special focus on attention, learning and cognition, motivation and reward, as well as response to stress and social interaction [6]. TAK-041 is an orally available, small-molecule GPR139 agonist. In animal models of social deficit, TAK-041 was shown to improve social interaction in a dose-dependent manner. Patients with schizophrenia have behavioural and cognitive deficits affecting social interactions. TAK-041 may, therefore, ameliorate negative symptoms of schizophrenia and be useful in the treatment of other disorders associated with social and cognitive dysfunction.

The development of a PET ligand suitable for GPR139 occupancy studies has been challenging, with the best available molecular candidates having affinity in the range of 10–50 nM, which may not be sufficient to provide an adequate signal-to-noise ratio for a PET ligand at this target [8–11]. Therefore, an alternative approach was employed in which a downstream effect of TAK-041 engaging with GPR139 was used as a pharmacodynamic biomarker.

Microdialysis studies in rodents have shown that pre-treatment with TAK-041 caused a significant attenuation of amphetamine (AMPH)-induced elevations of extracellular dopamine release in the nucleus accumbens, a brain region critical for cognitive processing of aversion, motivation, pleasure, reward and reinforcement learning [12]. Molecular imaging techniques using dopamine-D2 receptor (D2R) radioligands such as [^11^C]raclopride and [^123^I]IBZM have been shown to be suitable for the detection of pharmacologically induced dopamine release in healthy non-human primates [13, 14] and the human brain [15, 16]. Depletion of synaptic dopamine with α-methyl-para-tyrosine produces PET and single-photon emission computed tomography signal changes in the opposite direction to that produced by dopamine-enhancing drugs, such as AMPH or levo-DOPA [17–19]. The magnitude of the change in signal is related to the magnitude of the change in dopamine, as measured by microdialysis [20, 21], and differences between patients with neuropsychiatric disorders and healthy individuals have been demonstrated [20, 22, 23]. The utility of this methodology has been enhanced by the development of D2R agonist radioligands, such as [^11^C]PHNO [24, 25] and [^11^C]NPA [26]. As opposed to antagonists such as [^11^C]raclopride, [^123^I]IBZM, [^18^F]fallypride and [^11^C]FLB-457 that bind to the whole population of D2R, agonists bind primarily to the G-protein-coupled (G-coupled) state of the D2R, analogous to dopamine itself. Owing to this difference, agonist ligands are significantly more sensitive to fluctuations in synaptic dopamine concentration [27, 28]. A direct comparison of [^11^C]PHNO and [^11^C]raclopride demonstrated a 50–100% higher signal for [^11^C]PHNO in the healthy human brain [29].

In the present study, we translated the observation of blunted AMPH-induced dopamine release by TAK-041 in microdialysis experiments in rats, into PET studies in humans using [^11^C]PHNO as a non-invasive method of monitoring synaptic dopamine levels. Conducted as part of a phase 1 development plan, this study is an example of the use of PET imaging as a pharmacodynamic biomarker, reflecting a biological effect of the investigational compound downstream from target engagement.

## Methods

### Study design and participants

Twelve healthy male volunteers (39.8 ± 11.4 years of age) were screened and recruited into the study by Hammersmith Medicines Research, London. All imaging assessments were conducted at the Imanova Centre for Imaging Sciences (now Invicro), London, UK. The study was approved by the London—Brent Research Ethics Committee (reference 16/LO/1493), and permission to administer radioisotopes was obtained from the Administration of Radioactive Substances Advisory Committee of the UK (ref: 630/3764/35313). All study participants provided written informed consent prior to being enroled into the study.

**Fig. 1:**
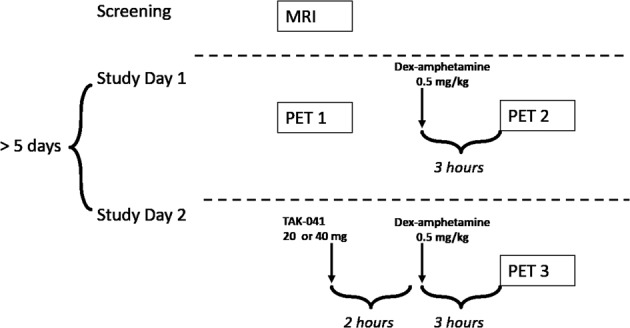
Schematic of study design. MRI magnetic resonance imaging, PET positron emission tomography.

Each participant underwent three [^11^C]PHNO PET scans, one at baseline, the second ~3 h after a single oral dose of d-AMPH (0.5 mg/kg), and the third ~5 h after a single oral dose of TAK-041 (20 mg or 40 mg) and 3 h after an oral dose of d-AMPH (0.5 mg/kg) (Fig. 1). There was a gap of >5 days between Study Day 1 and Study Day 2 to minimise any carry-over effects. The long plasma half-life of TAK-041, between 210 and 296 h following a single dose in healthy volunteers (data no shown), precluded the possibility to randomise the order of the post-d-AMPH scans.

Two participants received only a baseline PET scan owing to radioligand production failure at the post-dose scans. Data from these participants were not included in analyses.

### Magnetic resonance imaging

All participants had magnetic resonance imaging (MRI) scans (including a T1-weighted scan) at screening, performed using a Siemens 3T MRI Verio or Trio magnet (Siemens Healthcare, Erlangen, Germany). Structural imaging data were acquired in the sagittal plane, using a 3D magnetisation-prepared rapid gradient-echo scan with the following parameters: repetition time = 2300 ms, echo delay time = 2.98 ms, flip angle = 9°, isotropic voxels = 1.0 mm × 1.0 mm × 1.0 mm, number of slices = 160, total scanning time = 5 min 3 s. MRI scans were reviewed by a neuroradiologist to exclude the presence of any clinically relevant brain abnormalities in the study participants. T1 MRI data were used as part of the PET data analysis as described below.

### PET methods

#### [^11^C]PHNO preparation

[^11^C]PHNO was made as previously described [1, 25] by the reaction of [^11^C]-propionyl chloride with despropyl-PHNO (ABX, Radeberg, Germany). The final product was purified by high-performance liquid chromatography (HPLC) and subsequently reformulated through solid- phase extraction in a solution of 10% ethanol in normal saline.

#### PET data acquisition

Participants were positioned in the PET scanner, after the insertion of a venous cannula in an antecubital or forearm vein. Soft padding and head restraints were used to minimise head movement during data acquisition. All dynamic [11C]PHNO PET scans were performed on Siemens Biograph 6 PET/CT scanners (Hi-Rez [PET/CT1]) or a TruePoint with TrueV (Siemens Healthcare, Erlangen, Germany). All scans for a particular participant were performed using the same scanner. A low-dose CT scan was performed immediately before each PET study in order to estimate signal attenuation. After an intravenous bolus injection of [11C]PHNO, dynamic emission data were acquired for 90 min in 26 frames of increasing duration. The dynamic images were reconstructed using Fourier rebinning and a 2D-filtered discrete, inverse Fourier transform algorithm with a 5 mm isotropic Gaussian filter on a 128 × 128 matrix with 2.6 zoom giving 2 mm isotropic voxels. Appropriate corrections were applied for attenuation, randoms and scatter.

### Analysis methods

All image data were analysed using the MIAKAT^TM^ software package [30] (version 4.2.6.1), implemented using MATLAB (version R2016a; The MathWorks Inc., Natick, MA, USA), and using FSL (version 5.0.4; FMRIB, Oxford, UK [31, 32]) functions for brain extraction and SPM12 (Wellcome Trust Centre for Neuroimaging, London, UK; http://www.fil.ion.ucl.ac.uk/spm) for image segmentation and registration.

#### Image processing

Each participant’s structural magnetic resonance (MR) image underwent brain extraction and grey-matter segmentation and was co-registered to a standard reference space (Montreal Neurosciences Institute 152 [MNI152]; [33]). The MNI152 template brain image and associated atlas (CIC atlas; [34]) was non-linearly warped to the participant’s MR image to enable automated definition of regions of interest (ROIs).

The primary set of ROIs defined was the ventral striatum (VSt), putamen (Pu) (Fig. S1, Supplementary Information) and cerebellum. Putamen and ventral striatum were selected a priori based on a previous study from our group that found the most reproducible and robust reductions in [^11^C]-(+)-PHNO binding potential relative to the non-displaceable component (BP_ND_) after a d-AMPH challenge in these ROIs [29]. The cerebellum (Cer) was used as a reference region. An additional set of ROIs, including the caudate nucleus (Ca), globus pallidus (GP) and substantia nigra (SN), was also evaluated.

Dynamic PET images were registered to each participant’s MRI scan and corrected for motion using a frame-to-frame registration process with a normalised mutual information cost function. ROIs defined on the MRI images were applied to the dynamic PET data to derive regional time–activity curves (TACs).

#### Kinetic modelling

The simplified reference tissue model (SRTM) [35] has been demonstrated to be suitable for modelling [^11^C]PHNO PET data [1, 28, 34, 36, 37]. Regional TAC data extracted from the PET images were fitted using a basis function implementation of the SRTM [38] to estimate the BP_ND_ for each PET scan, as a measure of specific binding [39].

Reduction in [^11^C]PHNO-specific binding after a d-AMPH challenge $\left( {{\Delta}BP_{ND}^{AMPH}} \right)$ was quantified as per cent change in post-dose BP_ND_ $\left( {BP_{ND}^{AMPH}} \right)$from baseline BP_ND_ $\left( {BP_{ND}^{BL}} \right)$:

1 $${\Delta}BP_{ND}^{AMPH} \,=\, 100 \,\times\, \left( {1 \,-\, \frac{{BP_{ND}^{AMPH}}}{{BP_{ND}^{BL}}}} \right)$$

Reduction in [^11^C]PHNO-specific binding after administration of TAK-041 post-d-AMPH challenge $\left( {{\Delta}BP_{ND}^{AMPH \,+\, TAK - 041}} \right)$ was quantified as per cent change in post-dose BP_ND_ $\left( {BP_{ND}^{AMPH \,+\, TAK - 041}} \right)$from baseline BP_ND_ $\left( {BP_{ND}^{BL}} \right)$:

2 $${\Delta}BP_{ND}^{AMPH \,+\, TAK - 041} \,=\, 100 \,\times\, \left( {1 \,-\, \frac{{BP_{ND}^{AMPH \,+\, TAK - 041}}}{{BP_{ND}^{BL}}}} \right)$$

The effect of TAK-041 (ΔΔ*BP*_*ND*_) was quantified as the per cent change in ${\Delta}BP_{ND}^{AMPH \,+\, TAK - 041}$ compared with ${\Delta}BP_{ND}^{AMPH}$:

3 $${{{{{{{\mathrm{{\Delta}}}}}}}{\Delta}}}BP_{ND} \,=\, 100 \,\times\, \left( {1 \,-\, \frac{{{\Delta}BP_{ND}^{AMPH \,+\, TAK - 041}}}{{{\Delta}BP_{ND}^{AMPH}}}} \right)$$

All statistical comparisons were conducted using Student’s *t* tests for each ROI, using paired tests for within-participant comparisons and unpaired tests for between-group comparisons.

#### Plasma concentration of TAK-041

Venous blood samples for pharmacokinetic (PK) analysis of TAK-041, d-AMPH and l-AMPH were collected at the time of the post-dose PET scans into chilled blood-collection tubes (Vacutainer, Becton, Dickinson and Co., Franklin Lakes, NJ, USA) containing the anticoagulant ethylenediamine tetraacetic acid (K_2_EDTA). Plasma concentrations of TAK-041 were measured by HPLC with tandem mass spectrometry (MS) over a concentration range of 1–1500 ng/mL. Plasma concentrations of d-AMPH and l-AMPH were measured by HPLC with tandem MS over a concentration range of 0.1–100 ng/mL respectively.

## Results

Image data were successfully acquired for 32 PET scans in 12 study participants (see Table 1 for dose and image acquisition data). Two participants had only baseline [^11^C]PHNO PET scans acquired owing to radioligand synthesis failure. Each of the remaining 10 participants underwent three [^11^C]PHNO PET scans. Radiochemical purity for all scans was >95%, with a mean injected activity of 120 ± 26 MBq, and a mean injected PHNO mass of 18.4 ± 3.0 ng/kg. The maximal difference in injected mass for the three scans for each participant was <5%. See Table S1 in Supplementary Information for individual scan data.

**Table 1:** BP_ND_ data for individual participants.

Subject	Scan	Treatment	BP_ND_				
			Ca	Pu	VSt	SN	GP
1	PET1	None	1.22	2.15	2.49	1.12	1.89
	PET2	d-AMPH 0.5 mg/kg	1.08	1.77	1.85	0.66	1.54
	PET3	TAK-041 20 mg + d-AMPH 0.5 mg/kg	0.94	1.87	1.99	0.99	1.49
2	PET1	None	1.47	2.17	2.53	1.04	2.41
	PET2	d-AMPH 0.5 mg/kg	1.15	1.60	1.57	0.70	2.01
	PET3	TAK-041 20 mg + d-AMPH 0.5 mg/kg	1.17	1.65	1.72	0.67	1.89
3	PET1	None	1.70	2.29	2.65	0.99	1.93
	PET2	d-AMPH 0.5 mg/kg	1.29	1.61	1.96	0.75	1.55
	PET3	TAK-041 20 mg + d-AMPH 0.5 mg/kg	1.27	1.62	1.91	0.64	1.57
4	PET1	None	0.98	2.00	2.20	0.97	2.03
	PET2	d-AMPH 0.5 mg/kg	0.84	1.56	1.80	0.72	1.69
	PET3	TAK-041 20 mg + d-AMPH 0.5 mg/kg	0.92	1.64	1.92	0.84	1.70
5	PET1	None	1.59	2.24	2.44	1.02	2.55
	PET2	d-AMPH 0.5 mg/kg	1.32	1.74	1.97	0.85	2.14
	PET3	TAK-041 20 mg + d-AMPH 0.5 mg/kg	1.38	1.88	2.02	0.95	2.23
6	PET1	None	1.63	2.17	2.75	1.36	2.37
	PET2	d-AMPH 0.5 mg/kg	1.31	1.64	1.67	0.70	2.02
	PET3	TAK-041 40 mg + d-AMPH 0.5 mg/kg	1.44	1.82	1.76	0.92	2.37
7	PET1	None	0.57	1.69	2.35	1.06	2.41
	PET2	d-AMPH 0.5 mg/kg	0.49	1.27	1.61	0.77	2.05
	PET3	TAK-041 40 mg + d-AMPH 0.5 mg/kg	0.52	1.39	1.82	0.86	2.15
8	PET1	None	0.90	2.06	2.39	1.28	2.53
	PET2	d-AMPH 0.5 mg/kg	0.82	1.82	1.93	0.93	1.91
	PET3	TAK-041 40 mg + d-AMPH 0.5 mg/kg	0.85	1.93	2.04	1.31	2.18
9	PET1	None	1.55	2.19	2.72	1.43	2.12
	PET2	d-AMPH 0.5 mg/kg	1.44	1.82	2.10	1.16	2.20
	PET3	TAK-041 40 mg + d-AMPH 0.5 mg/kg	1.42	1.93	2.10	1.04	2.12
10	PET1	None	1.09	1.80	2.23	0.80	1.98
	PET2	d-AMPH 0.5 mg/kg	0.89	1.43	1.68	0.69	1.56
	PET3	TAK-041 40 mg + d-AMPH 0.5 mg/kg	0.95	1.59	1.97	0.77	1.82

*d-AMPH* d-amphetamine, *BP*_*ND*_ binding potential relative to the non-displaceable component, *Ca* caudate nucleus, *GP* globus pallidus, *PET* positron emission tomography, *Pu* putamen, *SN* substantia nigra, *VSt* ventral striatum.

**Fig. 2:**
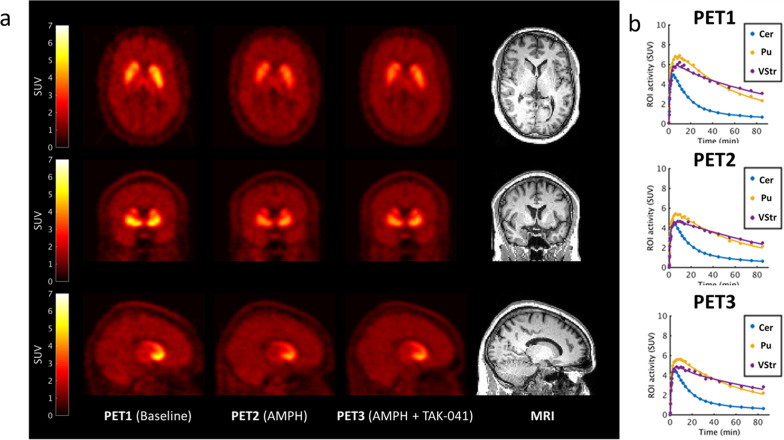
Brain images and time activity curves. **a** Orthogonal cross-sections of co-registered PET and MR images from a representative participant who received TAK-041 40 mg. For columns from left to right, images are PET1 (baseline), PET2 (post d-AMPH), PET3 (post d-AMPH + TAK-041), and structural (T1-weighted) MRI. PET images are shown as SUV summed from 10 to 90 min; **b** Time–activity curves and simplified reference tissue model fits (using cerebellum as reference region) for a representative participant (participant 10). AMPH amphetamine, Cer cerebellum, MRI magnetic resonance imaging, PET positron emission tomography, SUV standard uptake value, Pu putamen, ROI region of interest, VSt ventral striatum.

The PET images acquired displayed the expected anatomically heterogeneous signal, consistent with both the known distribution of D2/D3 receptors and previous [^11^C]PHNO data (Fig. 2).

AMPH and TAK-041 dose and plasma concentration immediately prior to [^11^C]PHNO administration for each individual, and the descriptive statistics for the plasma PK parameters of TAK-041, are detailed in Supplementary Information (Tables S2, S3). The TAK-041 exposure (based on AUC_∞_) was approximately twofold higher in the 40 mg than the 20 mg group, while the single time-point plasma concentrations at the start and end of the PET scan were between 1.4- and 1.5-fold higher. In general, the plasma concentrations of TAK-041, d-AMPH or l-AMPH were relatively stable over the course of the PET scans.

The SRTM produced good model fits to the tissue TAC data for all scans (Fig. 2) and BP_ND_ values were well determined (<10% CV) for the main ROIs. Calculated BP_ND_ values for all ROI are provided in Table 1. The BP_ND_ values for the primary ROIs are plotted in Fig. S2, Supplementary Information.

${\Delta}{{{{{{{\mathrm{BP}}}}}}}}_{{{{{{{{\mathrm{ND}}}}}}}}}^{{{{{{{{\mathrm{AMPH}}}}}}}}}$ values (effect of d-AMPH on [^11^C]PHNO BP_ND_) were calculated for each ROI for PET2 (d-AMPH-only scan), and ${\Delta}{{{{{{{\mathrm{BP}}}}}}}}_{{{{{{{{\mathrm{ND}}}}}}}}}^{{{{{{{{\mathrm{AMPH}}}}}} \,+\, {{{{{\mathrm{TAK - 041}}}}}}}}}$ values (effect of d-AMPH and TAK-041) were calculated for each ROI for PET3 (the d-AMPH + TAK-041 scan). The effect of pre-dosing with TAK-041 on the d-AMPH challenge, ΔΔBP_ND_, (the attenuation of ΔBP_ND_ by TAK-041) was determined for each region (see Table 2 for group means, and Table S4 in Supplementary Information for individual participants data).

**Table 2:** Effect of d-AMPH on [^11^C]PHNO BP_ND_ and modulation of this effect by TAK-041 pre-treatment.

**(a)**	**Mean (SD)** ${\Delta}{{{{{{{\mathrm{BP}}}}}}}}_{{{{{{{{\mathrm{ND}}}}}}}}}^{{{{{{{{\mathrm{AMPH}}}}}}}}}$**(%)**				
	**Pu**	**VSt**	**Ca**	**SN**	**GP**
All participants (*N* = 10)	21.6 (5.1)^1^	26.5 (7.7)^1^	15.6 (5.7)^1^	27.8 (10.9)^1^	15.9 (7.7)^1^
TAK-041 20 mg group (*n* = 5)	23.6 (4.6)^1^	25.4 (8.0)^1^	17.8 (5.7)^1^	28.4 (9.0)^1^	17.6 (1.5)^1^
TAK-041 40 mg group (*n* = 5)	19.6 (5.3)^1^	27.6 (8.2)^1^	13.4 (5.3)^1^	27.2 (13.6)^1^	14.2 (11.1)^1^

**(b)**	**Mean (SD) ΔΔBP**_**ND**_ **(%)**				
	**Pu**	**VSt**	**Ca**	**SN**	**GP**
All participants (*N* = 10)	26.4 (13.9)^2^	18.2 (17.1)^2^	10.3 (45.9)	33.2 (51.8)^2^	31.7 (44.6)
TAK-041 20 mg group (*n* = 5)	16.4 (11.3)^2, 3^	13.8 (14.1)^2^	−4.2 (59.2)	25.6 (49.7)	−3.2 (19.7)^3^
TAK-041 40 mg group (*n* = 5)	36.4 (7.7)^2, 3^	22.6 (20.3)	24.8 (26.7)	40.8 (58.6)	66.6 (32.4)^3^

*d-AMPH* d-amphetamine, *BP*_*ND*_ binding potential relative to the non-displaceable component, ${{{{{{{\mathrm{BP}}}}}}}}_{{{{{{{{\mathrm{ND}}}}}}}}}^{{{{{{{{\mathrm{AMPH}}}}}}}}}$ BP_ND_ after a d-AMPH challenge, *Ca* caudate nucleus, *GP* globus pallidus, *Pu* putamen, *SD* standard deviation, *SN* substantia nigra, *VSt* ventral striatum.

^1^Indicates a significant difference (*p* < 0.05) on a two-tailed paired *t* test, comparing baseline and post-amphetamine BP_ND_ for each participant.

^2^Indicates significant difference (*p* < 0.05) on a two-tailed, paired *t* test comparing ${\Delta}{{{{{{{\mathrm{BP}}}}}}}}_{{{{{{{{\mathrm{ND}}}}}}}}}^{{{{{{{{\mathrm{AMPH}}}}}}}}}$ and ${\Delta}{{{{{{{\mathrm{BP}}}}}}}}_{{{{{{{{\mathrm{ND}}}}}}}}}^{{{{{{{{\mathrm{AMPH}}}}}} \,+\, {{{{{\mathrm{TAK - 041}}}}}}}}}$ for each participant.

*d-AMPH* d-amphetamine, *BP*_*ND*_ binding potential relative to the non-displaceable component, *Ca* caudate nucleus, *GP* globus pallidus, *Pu* putamen, *ROI* region of interest, *SD* standard deviation, *SN* substantia nigra, *VSt* ventral striatum.

^3^Indicates significant difference (*p*  < 0.01) on an unpaired one-tailed *t* test comparing ΔΔBP_ND_ in each ROI between the 20 and 40 mg groups.

Administration of d-AMPH led to a significant reduction in BP_ND_ $\left( {{\Delta}{{{{{{{\mathrm{BP}}}}}}}}_{{{{{{{{\mathrm{ND}}}}}}}}}^{{{{{{{{\mathrm{AMPH}}}}}}}}}} \right)$ in all ROIs examined. There was no difference in ${\Delta}{{{{{{{\mathrm{BP}}}}}}}}_{{{{{{{{\mathrm{ND}}}}}}}}}^{{{{{{{{\mathrm{AMPH}}}}}}}}}$between the 20 and 40 mg groups. There was no relationship between the measured plasma concentration of d-AMPH and ΔBP_ND_ (See Fig. S3 in Supplementary Information).

**Fig. 3:**
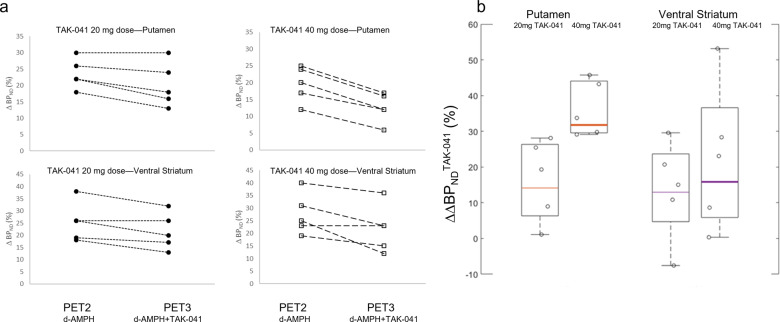
TAK-041 induced attenuation of dopamine release. **a** ΔBP_ND_ values in the putamen and ventral striatum for all participants; **b** box-and-whisker plot and individual ΔΔBP_ND_ values for putamen and ventral striatum for all participants. d-AMPH d-amphetamine, BP_ND_ binding potential relative to the non-displaceable component, PET positron emission tomography, ΔBP_ND_ change in BP_ND_ post-d-AMPH with positive values indicating reduction in BP_ND,_ ΔΔBP_ND_ the change in ΔBP_ND_ with positive values indicating a reduction in ΔBP_ND_ post- d-AMPH + TAK-041 compared to post-d-AMPH alone.

Pre-treatment with TAK-041 attenuated the effect of the d-AMPH challenge in all ROIs examined (Fig. 3). This effect was significant in the putamen and ventral striatum, as well as in the substantia nigra (Table 2). A dose effect of TAK-041 was observed in the putamen and globus pallidus, in which the ΔΔBP_ND_ in the 40 mg group was significantly greater than that in the 20 mg group (one-tailed *t* tests, *p* < 0.01). There was no relationship between the individual plasma concentration of TAK-041 and the ${{{{{{{\mathrm{{\Delta}}}}}}}{\Delta}}}BP_{ND}$ (Figs. S3, S4 in Supplementary Information).

## Discussion

The aim of this study was to evaluate the effects of TAK-041, a novel GPR139 agonist, on AMPH-induced dopamine release in the CNS, and indirectly comment on its brain penetration and target engagement. We have demonstrated a dose-dependent modulation of dopamine release in the human brain by TAK-041. Administration of d-AMPH produced a robust reduction of [^11^C]PHNO BP_ND_, consistent with an increase in synaptic dopamine concentration. As hypothesised, based on microdialysis data from studies in rodents, pre-treatment with TAK-041 reduced the magnitude of post-d-AMPH [^11^C]PHNO ∆BP_ND_, consistent with a reduced extracellular release of dopamine post d-AMPH administration. The plasma concentration of d-AMPH was similar in PET2 (d-AMPH-alone PET scan) and PET3 (d-AMPH + TAK-041 PET scan); hence, the attenuation of ${\Delta}{{{{{{{\mathrm{BP}}}}}}}}_{{{{{{{{\mathrm{ND}}}}}}}}}^{{{{{{{{\mathrm{AMPH}}}}}}}}}$ seen in PET3 probably reflects the pharmacological effects of TAK-041. The study design we used required some assumptions to be made. First, we assumed that the [^11^C]PHNO BP_ND_ returned to baseline levels 5 days after the administration of d-AMPH, in time for the TAK-041 scan day. We feel that this is a safe assumption based on the time-course of the d-AMPH effect measured in rats (using [^11^C]raclopride but applicable to other radioligands), with BP_ND_ returning to baseline levels between 8 and 24 h post-dose [40]. We also assumed that TAK-041 does not occupy D2/D3R at the doses administered, and thus has direct occupancy effects. TAK-041 demonstrated no significant effects when tested at 10μM concentration in a Eurofin Panlabs (Taipei, Taiwan) screen of 78 human class A, B, and C GPCRs, neurotransmitter receptors, ion channels, transporters, and enzymes, including D1-4Rs and DAT, consistent with our assumption (unpublished data). Second, we assumed that there is no reduction in the magnitude of dopamine release following a second dose of d-AMPH. Although the evidence is sparse, the available data indicates that repeated d-AMPH administration may lead to an *increase* in the dopamine released after multiple d-AMPH administrations (so called sensitisation—[41]), and little if any effect following a single dose [29]. Thus, we are confident that the reduction in the ΔBP_ND_ post-TAK-041 is not explained by a reduction in the effects of d-AMPH.

For all participants, TAK-041 induced a statistically significant attenuation of ${\Delta}{{{{{{{\mathrm{BP}}}}}}}}_{{{{{{{{\mathrm{ND}}}}}}}}}^{{{{{{{{\mathrm{AMPH}}}}}}}}}$ in the putamen and ventral striatum, the pre-defined ROIs. The changes seen in the secondary ROIs (the caudate, substantia nigra and the globus pallidus) followed the same pattern as those in the putamen and ventral striatum, although these did not always reach statistical significance. Our previous study examined the effect of d-AMPH administration on [^11^C]PHNO BP_ND_ and demonstrated that the most robust effects were seen in the putamen and ventral striatum [29]. The ventral striatum also contains the human equivalent of the rodent nucleus accumbens, the region in which TAK-041-induced attenuation of dopamine release was seen using microdialysis. We did not have the data for within-subject variability of the d-AMPH effect on [^11^C]PHNO BP_ND_; therefore, we used the between-subject-variability [29], as a worst-case scenario, to power this study and enable us to detect effects in the primary ROI of the putamen and ventral striatum. The lack of statistical significance in all the secondary regions in this relatively small study may, therefore, be attributed to low power, rather than a lack of effect. The modulation induced by the 40 mg dose of TAK-041 was greater than that induced by the 20 mg dose in all regions examined, the dose effect reaching statistical significance in the putamen and the globus pallidus. The lack of significant dose effects in other regions may therefore be due to the small sample size. Despite the dose effects, we saw no relationship between the individual plasma concentration of TAK-041 and ${{{{{{{\mathrm{{\Delta}}}}}}}{\Delta}BP}}_{{{{{{{{\mathrm{ND}}}}}}}}}$, possibly due to the small sample size as well as the limited range of plasma concentrations seen in this study (an ~1.4- to 1.5-fold difference between plasma concentrations in the 40 mg compared to 20 mg group).

This study was conducted using a fixed-order design, in which the post-AMPH PET scan always preceded the scan performed after administration of TAK-041-and AMPH. This design was dictated by the long plasma half-life of TAK-041, between 210 and 296 h following a single dose in healthy volunteers (data not shown), which made randomisation of the scan order impractical; however, it opens up the possibility of order effects influencing the study results. The presence of dose-dependent effects (with TAK-041 40 mg producing greater effects than the 20 mg dose) is consistent with a pharmacological effect by TAK-041. Replication in a larger counterbalanced design study would be required to confirm this.

The mechanism underlying the modulation of dopamine release by TAK-041 is unclear. Amphetamine effects are independent of neurone firing rate, and therefore the effects of TAK-041 are most likely to be expressed in either modulating dopamine synthesis, modulating dopamine packing in synaptic vesicles, affecting vesicle fusion with the plasma membrane, or interacting with the cell membrane dopamine transporter reabsorbing synaptic dopamine into the pre-synaptic terminal. We have no evidence for GPR139 ligands interacting directly with either synaptic vesicles or the dopamine transporters. However GPR139 expression was shown to correlate with D2R expression in a range of brain regions, and the two receptors were shown to interact in a stable cell line [7]. A similar interaction was demonstrated with MC_3_ and MC_5_ receptors [42] and μ-opioid receptors [43], indicating that GPR139 can heterodimerize with other G-protein coupled receptors and modulate their signalling. Thus, a modulation of D2R autoreceptors by TAK-041 to reduce dopamine synthesis and hence synaptic dopamine release may be one plausible mechanism to ameliorate the effects of amphetamine, though the rapid action (within several hours of administration) is a factor to consider. The endogenous ligand for GPR139 has not been elucidated, with the amino acids l-tryptophan and l-phenylalanine demonstrating affinity in the physiologically plausible micromolar range. However, the peptide hormones adrenocorticotropic hormone and α-melanocyte stimulating hormone (α-MSH) have demonstrated affinity in the high nanomolar range, and may also be plausible candidates [44]. The predicted cleavage product of pre-pro-protein pro-opiomelanocortin, α-MSH_1–10_, is the most potent and selective endogenous agonist of GPR139 identified to date (EC_50_ = 318 nM) [42]. The opioid/melanocortin system and the dopamine system have been shown to interact and mutually modulate each other’s neurotransmission. In preclinical models, GPR139 agonism has been shown to ameliorate compulsive alcohol drinking and hyperalgesia [45], as well as morphine-induced analgesia, reward and withdrawal [43], supporting the idea that GPR139 may have a physiological role in controlling brain dopamine release.

The high attrition in CNS drug development has prompted the search for ways to improve drug survival once they reach phase 2. The approach formulated by Morgan et al. has posited that a molecule for which the ‘three pillars of drug development’ have been demonstrated has a significantly higher probability of progressing to late-stage development [46]. PET imaging is uniquely placed to demonstrate drug distribution to the CNS and drug–target engagement in a physiologically meaningful dose range. PET studies quantifying the brain distribution of novel compounds labelled with PET radionuclides [47, 48], and determining the relationship between the concentration of the drug in plasma and CNS-target occupancy using selective PET radioligands [1, 2], have become a critical part of the development of novel medication. However, such studies require the availability of a well-characterised PET radioligand for the molecular target in question, or radiolabelling of the drug itself, an activity which may not always be practical. The demonstration of pharmacodynamic consequences of target engagement is a viable alternative in such situations. More specifically, an approach such as that used in this paper can be used if a radioligand sensitive to physiologically relevant endogenous neurotransmitter fluctuations is available.

Although the principles of PET-based evaluation of dopamine fluctuations in the living human brain are well established [16], this is the first demonstration of its utility in early-phase drug development. Modulation of AMPH-induced dopamine release in animal studies [49, 50] established the feasibility of this approach, but the changes found in [^11^C]raclopride and [^123^I]IBZM binding were considered to be too small and variable to detect reliable modulation by a novel drug. Agonist radioligands, such as [^11^C]PHNO, demonstrate a 50–100% greater signal change post-AMPH challenge [29], enabling the detection of modulatory effects of novel compounds. While the use of PET methods to detect dopamine fluctuations is well established, detection of change in other neurotransmitters has been more problematic. Nevertheless, recent developments have enabled the use of this approach for the opioid [51], acetylcholine [52] and serotonin [53] systems.

In conclusion, we used a novel application of established PET methodology to provide strong support for the hypothesis that TAK-041 crosses the blood–brain barrier and attenuates dopamine release in healthy human volunteers. GPR139 has been proposed to have a role as a therapeutic target in schizophrenia, addiction and metabolic disorders, and attention-deficit/hyperactivity disorder, as well as Parkinson’s disease and phenylketonuria [44]. The data presented here demonstrate that TAK-041 readily enters the brain and produces pharmacological effects consistent with a modulation of dopamine release, demonstrating the potential of TAK-041 as a novel treatment for a variety of neuropsychiatric disorders.

## Supplementary information

Supplementary Information - Revised/Clean
