# Supplementary material for: Endogenous dopamine release in the human brain as a pharmacodynamic biomarker: evaluation of the new GPR139 agonist TAK-041 with [11C]PHNO PET
Source: Neuropsychopharmacology. 2021 Oct 21;47(7):1405–12. doi: 10.1038/s41386-021-01204-1 (PMC9117280; doi:10.1038/s41386-021-01204-1)
Supplement: Supplementary file 1 — Supplementary Information - Revised/Clean [file 41386_2021_1204_MOESM1_ESM.docx]

# Supplementary Information

**Table S1.** Individual scan radiochemistry parameters.

| **Participant** | **Scan** | **Injected activity (MBq)** | **Injected mass (ng/kg)** | **Radiochemical purity (%)** |
| --- | --- | --- | --- | --- |
| 1 | PET1 | 110 | 18.1 | 100 |
|  | PET2 | 133 | 18.5 | 100 |
|  | PET3 | 98 | 17.8 | 100 |
| 2 | PET1 | 111 | 17.9 | 100 |
|  | PET2 | 109 | 19.1 | 100 |
|  | PET3 | 110 | 17.5 | 100 |
| 3 | PET1 | 122 | 13.7 | 100 |
|  | PET2 | 104 | 13.6 | 100 |
|  | PET3 | 130 | 14.1 | 100 |
| 4 | PET1 | 115 | 13.5 | 100 |
|  | PET2 | 129 | 13.3 | 100 |
|  | PET3 | 119 | 13.4 | 100 |
| 5 | PET1 | 118 | 18.2 | 100 |
|  | PET2 | 149 | 18.9 | 100 |
|  | PET3 | 146 | 17.9 | 100 |
| 6 | PET1 | 158 | 17.9 | 95.2 |
|  | PET2 | 119 | 18.7 | 100 |
|  | PET3 | 121 | 18.8 | 100 |
| 7 | PET1 | 146 | 17.9 | 95.2 |
|  | PET2 | 105 | 17.4 | 100 |
|  | PET3 | 107 | 17.6 | 100 |
| 8 | PET1 | 100 | 17.9 | 100 |
|  | PET2 | 150 | 17.9 | 100 |
|  | PET3 | 86 | 17.9 | 100 |
| 9 | PET1 | 67 | 23.0 | 100 |
|  | PET2 | 128 | 22.4 | 100 |
|  | PET3 | 177 | 22.9 | 100 |
| 10 | PET1 | 68 | 22.9 | 100 |
|  | PET2 | 119 | 22.1 | 100 |
|  | PET3 | 178 | 22.5 | 100 |
| 11 | PET1 | 100 | 21.2 | 100 |
| 12 | PET2 | 101 | 23.1 | 100 |

PET, positron emission tomography.

**Table S2**. d-AMPH and TAK-041 dose administered, and plasma concentration immediately prior to [^11^C]PHNO administration.

| **Participant** | **Scan** | **Plasma d-AMPH (ng/mL)** | **TAK-041 dose (mg)** | **Plasma TAK-041 (ng/mL)** |
| --- | --- | --- | --- | --- |
| 1 | PET1 | – | – | – |
|  | PET2 | 93.1 | – | – |
|  | PET3 | 91.5 | 20 | 321 |
| 2 | PET1 | – | – | – |
|  | PET2 | 53.2 | – | – |
|  | PET3 | 72.5 | 20 | 359 |
| 3 | PET1 | – | – | – |
|  | PET2 | 101.0 | – | – |
|  | PET3 | 90.7 | 20 | 289 |
| 4 | PET1 | – | – | – |
|  | PET2 | 63.9 | – | – |
|  | PET3 | 75.8 | 20 | 285 |
| 5 | PET1 | – | – | – |
|  | PET2 | 113.0 | – | – |
|  | PET3 | 91.6 | 20 | 265 |
| 6 | PET1 | – | – | – |
|  | PET2 | 95.0 | – |  |
|  | PET3 | 92.6 | 40 | 518 |
| 7 | PET1 | – | – | – |
|  | PET2 | 106.0 | – | – |
|  | PET3 | 114.0 | 40 | 545 |
| 8 | PET1 | – | – | – |
|  | PET2 | 65.0 | – | – |
|  | PET3 | 72.7 | 40 | 450 |
| 9 | PET1 | – | – | – |
|  | PET2 | 81.2 | – | – |
|  | PET3 | 88.2 | 40 | 319 |
| 10 | PET1 | – | – | – |
|  | PET2 | 81.2 | – | – |
|  | PET3 | 81.8 | 40 | 476 |

d-AMPH, d-amphetamine; PET, positron emission tomography.

**Table S3.** Summary of plasma pharmacokinetic parameters of TAK-041 after a single dose of TAK-041 20 mg or 40 mg administered as an oral suspension.

| **Dose (mg)** | ***n*** | **t_max_ (h)** | **C_max_ (ng/mL)** | **C_PET start_  (ng/mL)** | **C_PET end_ (ng/mL)** | **AUC_t_ (d*ng/mL)** | **AUC_∞_ (d*ng/mL)** | **t_½z_ (d)** |
| --- | --- | --- | --- | --- | --- | --- | --- | --- |
| 20 | 5 | 6.5  (1.0, 12.0) | 374 (16.8) | 304 (12.1) | 321 (17.0) | 3 898 (67.7) | 3 933 (67.9) | 9.66 (72.9) |
| 40 | 5 | 1.0  (1.0, 1.9) | 575 (23.9) | 462 (19.0) | 449 (15.0) | 7406 (24.5) | 7688 (27.0) | 11.60 (36.1) |

Values represent mean (%CV), except for t_max_ for which median (minimum, maximum) are presented. AUC_t_, area under the plasma concentration–time curve from time 0 to time t; AUC_∞_, area under the plasma concentration–time curve from time 0 to infinity; C_max_, maximum concentration; C_PET start_, concentration at the start of the PET scan; C_PET end_, concentration at the end of the PET scan; d, days; %CV, coefficient of variance expressed as percent; t_max_, time of maximum concentration; t_½z,_ terminal disposition phase half-life.

**Table S4**. ΔBP_ND_ and ΔΔBP_ND_ data.

| **Participant** | **Scan** | **ΔBP_ND_ (%)** | | | | | **ΔΔBP_ND_ (%)** | | | | | |
| --- | --- | --- | --- | --- | --- | --- | --- | --- | --- | --- | --- | --- |
|  |  | **Ca** | **Pu** | **VSt** | **SN** | **GP** | **Ca** | **Pu** | **VSt** | **SN** | **GP** |  |
| 1 | PET2 | 11 | 18 | 26 | 41 | 18 | – | – | – | – | – |  |
|  | PET3 | 23 | 13 | 20 | 11 | 21 | −103 | 25 | 21 | 73 | −15 |  |
| 2 | PET2 | 22 | 26 | 38 | 33 | 17 | – | – | – | – | – |  |
|  | PET3 | 20 | 24 | 32 | 36 | 22 | 7 | 9 | 15 | −8 | −30 |  |
| 3 | PET2 | 25 | 30 | 26 | 25 | 20 | – | – | – | – | – |  |
|  | PET3 | 25 | 30 | 26 | 25 | 20 | −3 | 1 | −8 | −44 | 6 |  |
| 4 | PET2 | 14 | 22 | 18 | 26 | 17 | – | – | – | – | – |  |
|  | PET3 | 7 | 18 | 13 | 14 | 16 | 53 | 19 | 30 | 47 | 2 |  |
| 5 | PET2 | 17 | 22 | 19 | 17 | 16 | – | – | – | – | – |  |
|  | PET3 | 13 | 16 | 17 | 7 | 13 | 25 | 28 | 11 | 60 | 21 |  |
| **20 mg group**  **Mean (SD)** | **PET2** | **17.8 (5.7)** | **23.6 (4.6)** | **25.4 (8.0)** | **28.4 (9.0)** | **17.6 (1.5)** | – | – | – | – | – |  |
|  | **PET3** | **17.6 (7.5)** | **20.2 (6.8)** | **21.6 (7.5)** | **18.6 (11.8)** | **18.4 (3.8)** | **28.3 (23.2)** | **16.4 (11.3)** | **13.8 (14.1)** | **25.6 (49.7)** | **-3.2 (19.7)** |  |
| 6 | PET2 | 19 | 24 | 40 | 49 | 14 | – | – | – | – | – |  |
|  | PET3 | 11 | 16 | 36 | 32 | 0 | 42 | 34 | 9 | 34 | 101 |  |
| 7 | PET2 | 14 | 25 | 31 | 27 | 15 | – | – | – | – | – |  |
|  | PET3 | 9 | 17 | 23 | 19 | 11 | 33 | 29 | 28 | 31 | 28 |  |
| 8 | PET2 | 9 | 12 | 19 | 28 | 25 | – | – | – | – | – |  |
|  | PET3 | 5 | 6 | 15 | −2 | 14 | 41 | 46 | 23 | 108 | 44 |  |
| 9 | PET2 | 7 | 17 | 23 | 19 | −4 | – | – | – | – | – |  |
|  | PET3 | 9 | 12 | 23 | 27 | 0 | −22 | 30 | 0 | -47 | 98 |  |
| 10 | PET2 | 18 | 20 | 25 | 13 | 21 | – | – | – | – | – |  |
|  | PET3 | 13 | 12 | 12 | 3 | 8 | 30 | 43 | 53 | 78 | 62 |  |
| **40 mg group**  **Mean (SD)** | **PET2** | **13.4 (5.3)** | **19.6 (5.3)** | **27.6(8.2)** | **27.2 (13.7)** | **14.2 (11.1)** | – | – | – | – | – |  |
|  | **PET3** | **9.4 (3.0)** | **12.6 (4.3)** | **21.8 (9.3)** | **15.8 (14.8)** | **6.6 (6.4)** | **36.5 (5.9)** | **36.4 (7.7)** | **22.6 (20.3)** | **40.8 (58.6)** | **66.6 (32.4)** |  |

ΔBP_ND_ was calculated as the percentage change in BP_ND_ after d-AMPH administration. ΔΔBP_ND_ was calculated as the relative percentage change in BP_ND_ in the AMPH + TAK-041 condition compared with the d-AMPH-alone condition. d-AMPH, d‑amphetamine; BPND, binding potential relative to the non-displaceable component;
Ca, caudate nucleus; GP, globus pallidus; PET, positron emission tomography; Pu, putamen; SN, substantia nigra; VSt, ventral striatum.


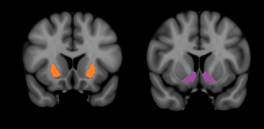


**Fig. S1** Representative T1 MRI showing putamen (left) and ventral striatum (right) region of interest placement.

**
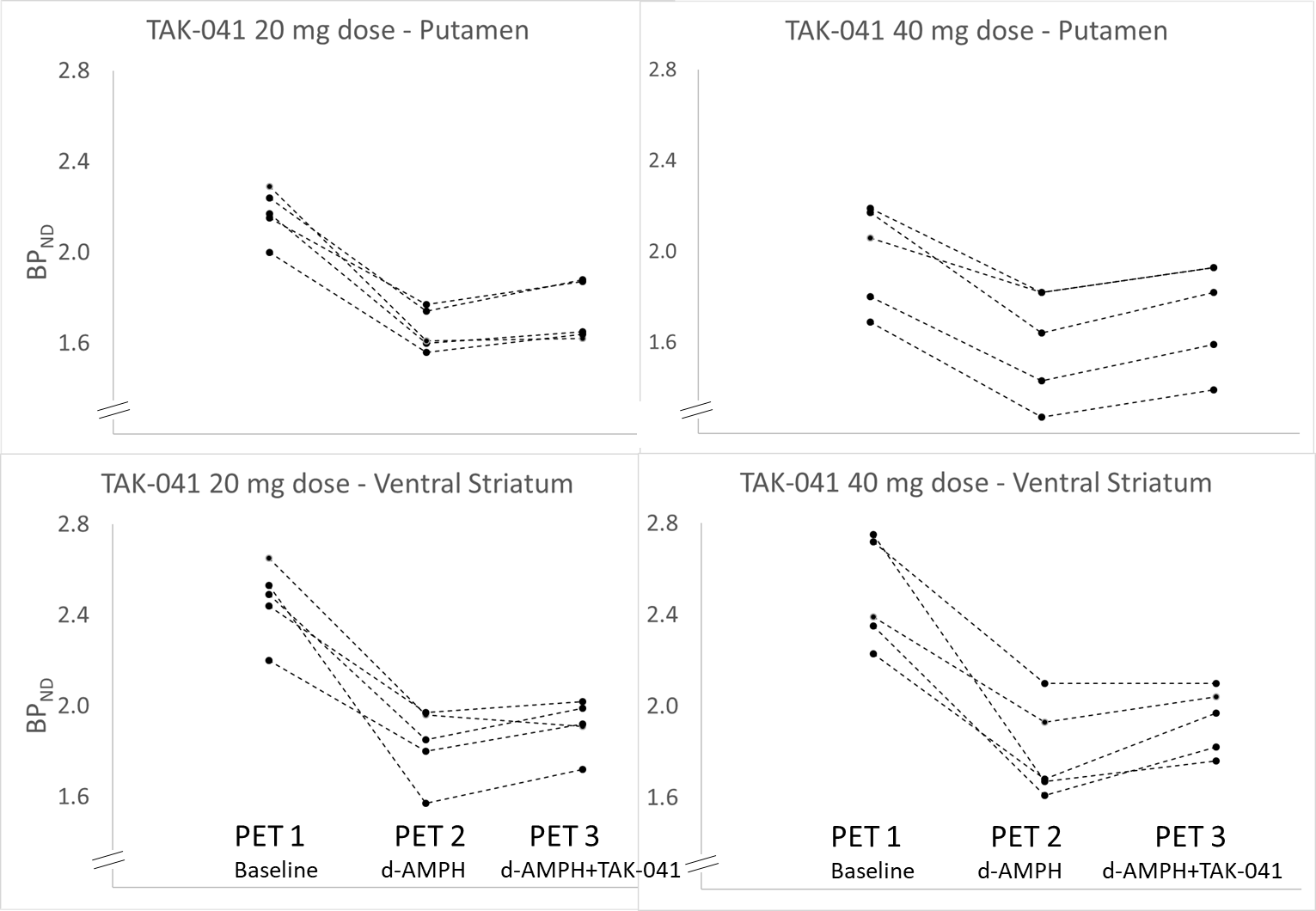
**

**Fig. S2** BP_ND_ values in the putamen and ventral striatum for all scans. d-AMPH, d-amphetamine; BP_ND,_ binding potential relative to the non-displaceable component; PET, positron emission tomography.


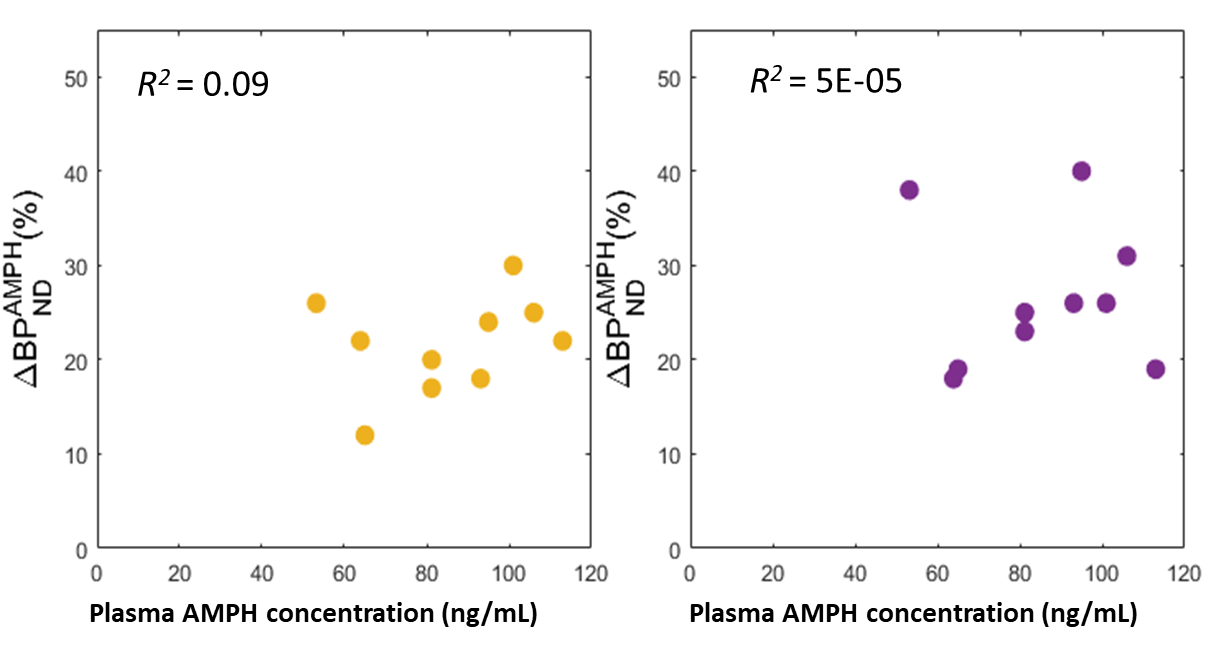


**Fig. S3.** Putamen (left plot) and ventral striatum (right plot) $\text{∆BP}_{\text{ND}}^{\text{AMPH}}$ values plotted against measured plasma concentration of AMPH (approximately 3 hours post AMPH administration). AMPH, amphetamine; BP_ND_, binding potential relative to the non-displaceable component; $\text{BP}_{\text{ND}}^{\text{AM}\text{P}\text{H}}$, BP_ND_ post AMPH administration.

R^2^= 5E-05

R^2^ = 0.09


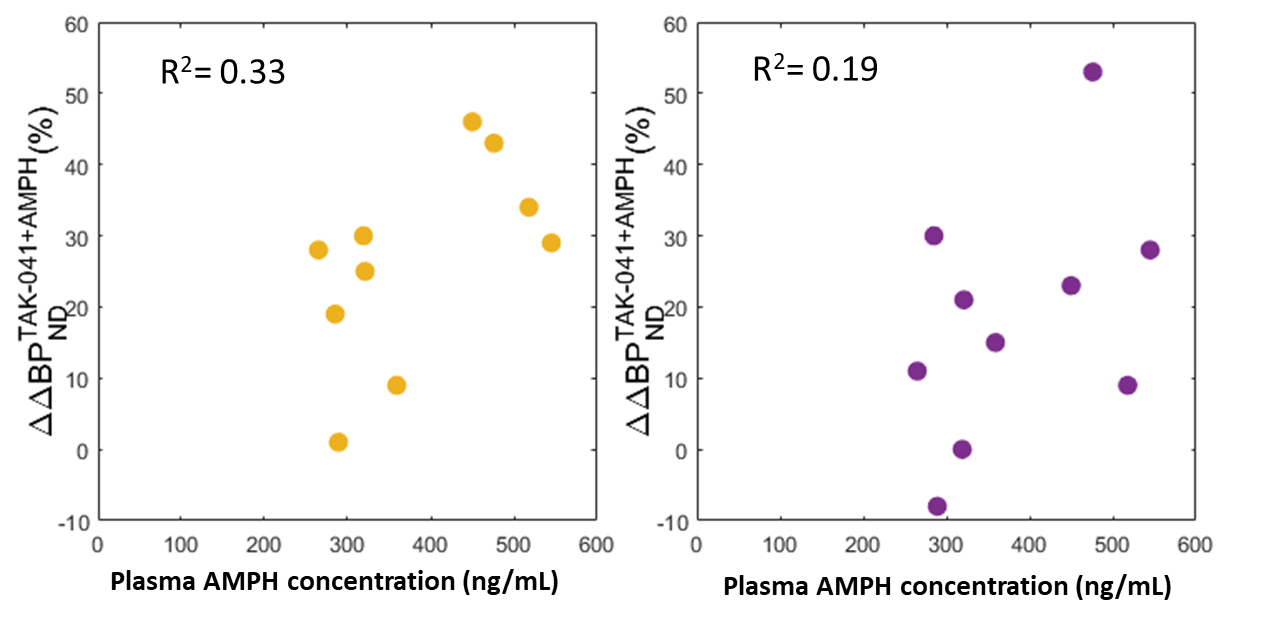


**Fig. S4.** Putamen (left plot) and ventral striatum (right plot). ∆∆BP_ND_ plotted against measured plasma concentration of TAK-041 (approximately 5 hours post TAK-041 administration). BP_ND_, binding potential relative to the non-displaceable component;$\text{ }\text{BP}_{\text{ND}}^{\text{TAK-041+AM}\text{P}\text{H}}$, BP_ND_ post amphetamine and TAK‑041 administration.
